# Stratification of Carbon Fractions and Carbon Management Index in Deep Soil Affected by the Grain-to-Green Program in China

**DOI:** 10.1371/journal.pone.0099657

**Published:** 2014-06-10

**Authors:** Fazhu Zhao, Gaihe Yang, Xinhui Han, Yongzhong Feng, Guangxin Ren

**Affiliations:** College of Agronomy, Northwest A&F University, Yangling, Shaanxi, China; and The Research Center of Recycle Agricultural Engineering and Technology of Shaanxi Province, Yangling, Shaanxi, China; Institute for Plant Protection (IPP), CNR, Italy

## Abstract

Conversion of slope cropland to perennial vegetation has a significant impact on soil organic carbon (SOC) stock in A horizon. However, the impact on SOC and its fraction stratification is still poorly understood in deep soil in Loess Hilly Region (LHR) of China. Samples were collected from three typical conversion lands, *Robinia psendoacacia* (RP), *Caragana Korshinskii Kom* (CK), and abandoned land (AB), which have been converted from slope croplands (SC) for 30 years in LHR. Contents of SOC, total nitrogen (TN), particulate organic carbon (POC), and labile organic carbon (LOC), and their stratification ratios (SR) and carbon management indexes (CMI) were determined on soil profiles from 0 to 200 cm. Results showed that the SOC, TN, POC and LOC stocks of RP were significantly higher than that of SC in soil layers of 0–10, 10–40, 40–100 and 100–200 cm (P<0.05). Soil layer of 100–200 cm accounted for 27.38–36.62%, 25.10–32.91%, 21.59–31.69% and 21.08–26.83% to SOC, TN, POC and LOC stocks in lands of RP, CK and AB. SR values were >2.0 in most cases of RP, CK and AB. Moreover, CMI values of RP, CK, and AB increased by 11.61–61.53% in soil layer of 100–200 cm compared with SC. Significant positive correlations between SOC stocks and CMI or SR values of both surface soil and deep soil layers indicated that they were suitable indicators for soil quality and carbon changes evaluation. The Grain-to-Green Program (GTGP) had strong influence on improving quantity and activity of SOC pool through all soil layers of converted lands, and deep soil organic carbon should be considered in C cycle induced by GTGP. It was concluded that converting slope croplands to RP forestlands was the most efficient way for sequestering C in LHR soils.

## Introduction

Soil organic carbon (SOC) is a dynamic component of the terrestrial system, with internal changes in both vertical and horizontal directions and external exchanges between the atmosphere and the biosphere [1]. SOC storage is estimated at approximately 1500 Pg globally, which is about two and three times the size of carbon pools in the atmosphere and vegetation, respectively [2]. Since carbon uptake and storage is tightly linked to the nitrogen (N) cycle, it is equally important to understand how N pools and fluxes are affected by land use change [3]. Moreover, more than 50% of the total SOC is stored in the subsoil [4]. The proportion of soil organic matter (SOM) stored in the first meter of the world soils below 30 cm depth ranges 46%∼63%, except for Podzoluvisols, where 30% of SOC is stored below the depth of 30 cm [4]. A recent study also suggests that in the northern circumpolar permafrost region, at least 61% of the total soil C is stored below 30 cm [5]. Therefore, subsoil C may be even more important in terms of source or sink for CO_2_ than topsoil C [6]. Considering the potential role of SOC in atmospheric CO_2_ sink, it is important to understand what leads to sequestration of large amounts of SOC in the subsoil or even in deep soil. However, the SOC contents in deep soil layers are not fully understood in LHR of China to date.

As an indicator of soil quality, SOM stratification, which is related to the rate and amount of SOC sequestration [7], is common in many natural ecosystems [8] and managed grasslands and forests [9]–[10]. Stratification ratio (SR) is defined as the ratio of a soil property at the surface layer to that at a deeper layer. In general, high SR values indicate good soil quality and are usually used to assess agricultural practices [7]. For instance, SR values for SOC at depths of 0–5 cm and 20–40 cm range from 1.1 to 1.5 under traditional tillage (TT) while from 1.6 to 2.6 under conservation tillage (CT) [11]. Little information is available on natural ecosystems and managed shrubs or forests land. Additionally, under semiarid climate, SOC in active fractions, such as particulate organic carbon (POC) and labile organic carbon (LOC) was more sensitive to soil management practices than total SOC [12]. Previous researches have indicated that changing rate of POC and LOC was faster than SOC in whole soil [11], and they could be an early indicator for SOC change in soil [13]. Meanwhile, the carbon management index (CMI), which is derived from the total soil organic C pool and C lability, had been extensively used as a sensitive indicator of SOC variation rate in response to soil management changes [14]–[15]. Therefore, under semi-arid climate, using SR of total SOC and of different SOC fractions may be useful to reveal how soil management affects soil quality and helpful to understand the mechanism of SOC transformation and cycling in subsoil as well as in deep soil. In LHR of China, soil erosion and desertification are causing a loss of net primary productivity that was estimated as high as 12 kg C ha^−1^y^−1^ [16]. To counteract soil erosion and other environmental problems, an environmental protection policy was implemented by Chinese central government, which was known as the Grain to Green Program (GTGP). The purpose of GTGP was to convert up to 26.87 million ha low-yield sloped croplands (>25°) into forests, shrubs or grasslands by the end of 2008 [17]. It is the first and the most ambitious “payment–for–ecosystem–services” program in China to date [18]. Although the initial goal of GTGP was to control soil erosion in China, it also plays a significant role in circulation of SOC and total nitrogen (TN). In recent years, a few studies estimated the effects of GTGP on vegetation structure, economic benefits, soil physiochemical properties, and niche characteristics [19]–[21]. However, SR values of SOC and/or TN and CMI value among different land use types are rarely reported. Especially, information on dynamics of C in deep soil is largely ignored in this region.

This study aimed to: 1) analyze the contents of SOC, TN, POC and LOC and their vertical distributions at the depths of 0–200 cm; 2) assess the stocks of SOC and TN at different soil depths of three land use types; and 3) evaluate the soil quality of different land use types using SR and CMI values as the main assessment parameters.

## Materials and Methods

All sites in the watershed we were selected for study was determined through interviews with local farmers (Mr. Yibin Zhang, Soil and Water Conservation Experiment Station, Northwest A&F University, Ansai County, Shaanxi, NW China).We state clearly that no specific permissions were required for the location. We confirm that the location is not privately-owned or protected in any way. We confirm that the field studies do not involve endangered or protected species.

### Research area

**Figure 1:**
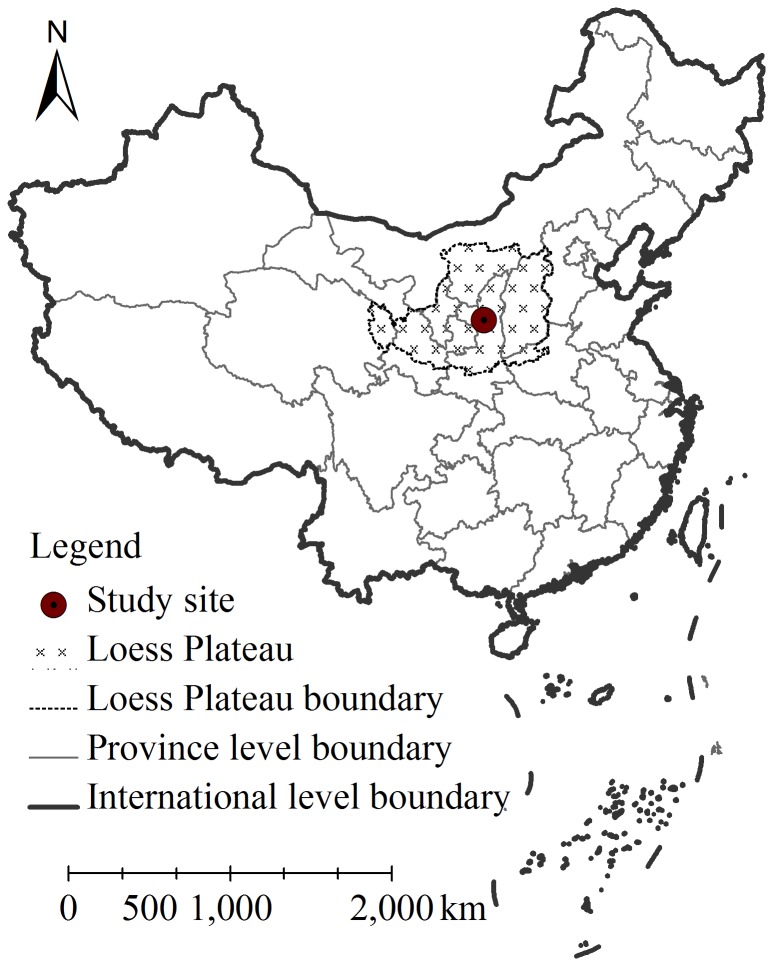
Location of the Loess Plateau and the study site.

The study was conducted in the Zhifanggou catchment (36°46′42″–36°46′28″N, 109°13′46″–109°16′03″E), which is located in Ansai county, central LHR (see Fig. 1). Ansai is a typical county characterized by semi-arid climate and hilly loess landscape in the Loess Plateau with an annual average temperature of 8.8°C, and an average annual precipitation of 505 mm. 60% of precipitation occurs between July and September (∼300 mm in dry years while >700 mm in wet years). Accumulated temperatures above 0°C and 10°C are 3733°C and 3283°C, respectively. On average, there are about 157 frost-free days and 2415 h sunny time each year. Arable farming mostly occurs on sloping lands without irrigation. The loess parent material at the site has an average thickness of approximately 50–80 m and the soil in this region is classified as Calciustepts soil [22]. Sand (2–0.05 mm) and silt (0.05–0.002 mm) account for approximately 29.22% and 63.56% in soil depth of 0–20 cm, respectively. The soil is highly erodible, with an erosion modulus of 10,000–12,000 Mg·km^−2^·yr^−1^ before the start of restoration efforts [23]. After 30 years vegetation restoration, the area of forest lands significantly increased from 5% to 40% [24].

The Zhifanggou catchment has been an experimental site of the Institute of Soil and Water Conservation, Chinese Academy of Science (CAS) since 1973 [25]. The major agricultural land use type in LHR is slope cropland. Agricultural management in this region, including the major crop types grown, has not been changed significantly since the 1970s. The main crops grown in these sites were millet (*Setaria italica*) and soybean (*Glycine max*) rotation, and no irrigation was provided in grown season (depend on rainfall). One crop was grown each year, and fertilizer was applied (mainly manure). After more than 30 years of comprehensive management, the ecological environment of the catchment has been significantly improved [26]. Since late 1970s, slope cropland is replanted with shrubs and woods, mainly *Robinia pseudoacacia L*. (RP) and *Caragana Korshinskii Kom (CK)*, to control soil erosion (see Table 1). Abandoned cropland was also generated during this period due to its extremely low productivity and long distance from farmers' residences [27]–[28]. Despite wild grasslands and shrub lands were usually found on steep slopes, these sites were used for firewood collection as well. So the wild vegetation was of limited coverage or even barren for long periods. In 1999, most slope lands were closed for vegetation restoration under the GTGP [29].

**Table 1:** Characteristics of different vegetation types.

Vegetation types	Age	Canopy closure (%)	Litter accumulation (t.ha^−2^)	Undergrowth Vegetation^a^	Species diversity indices
*Robinia pseudoacacia L.*	30	58	20.5	*Lespedeza dahurica - Stipa bungeana*	6.5
*Caragana Korshinskii Kom*	30	50	13.3	*Achillea capillaries, Stipa bungeana*	3.9

a means the main vegetation in forest/shrub land.

### Soil sampling

In September 2012, based on land use history, 30 year old *Robinia psendoacacia* (RP), *Caragana Korshinskii Kom* (CK), abandoned land (AB) and slope cropland (SC) in the Zhifanggou catchment were selected. Three 30 m×20 m plots were established for each land use type. All sites were located on the same physiographical units with same slope aspects, same elevation of 1250 m and a spatial distance of 1200 m.

Soil samples were taken at several soil depths using a soil auger (diameter 5 cm) from 10 points within “S” shape at each plot (0–10 cm, 10–20 cm, 20–30 cm, 30–40 cm, 40–50 cm, 50–60 cm, 60–70 cm, 70–80 cm, 80–90 cm, 90–100 cm, 100–120 cm, 120–140 cm, 140–160 cm, 160–180 cm, and 180–200 cm). Then after removing the litter layer, ten soil samples at each depth of each plot were mixed to make one sample. Samples were collected at least 80 cm away from the trees. All samples were sieved through a 2 mm screen, and roots and other debris were removed. Soil samples were air-dried and stored at room temperature for the determination of soil chemical properties. A ring tube was used to determine the bulk density in each soil depth.

### Laboratory analysis

SOC content (g.kg^−1^) and TN content (g.kg^−1^) were determined usingK_2_Cr_2_O_7_ oxidation method and Kjeldhal method, respectively [30].

To determine POC content, 25 g soil was dispersed with 100 mL of 5 g L^−1^ sodium hexametaphosphate before being. Then, the mixed soil solution was shaken for 1 h at high speed on an end-to-end shaker and screened by a 0.053 mm sieve with several deionized water rinses. The soil remained on the sieve was backwashed into a pre-weighed aluminum box and dried at 60°C for 24 h, then it was grounded for analysis of C [31].

Soil labile organic carbon (LOC) was measured following the method described in Graeme et al. [32]. A 2–6 g air dried soil sample was put into a 50 mL centrifuge tube, and 25 mL of 333 mmolL^−1^ KMnO_4_ solution was added before being shaken with a rate of 120 rpm for 1 h, and centrifuged for 5 min with a rate of 5,000×g. The upper clear solution was transferred, and diluted by 250 times, and then the absorbance at 565 nm wavelength was determined. The absorbances at values 565 nm with different KMnO_4_ concentrations were also determined for preparation of standard curve, which was used for the determination of the KMnO_4_ concentrations. Difference between the amounts of KMnO_4_ added and remained was used to calculate labile C concentration in the soil sample.

Calculation of SOC (TN) stocks, SR of SOC (TN, POC, and LOC) and CMI

SOC density (SOCD, TND) represents the total SOC (TN) storage of overall certain sampling depth. SOCD(TND) of different sampling depths were calculated: 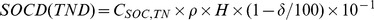
(1)where SOCD (TND) is the density (Mg·ha^−1^) of SOC (TN) and C_SOC,TN_ is the content (g·kg^−1^) of SOC (TN). ρ is the bulk density (g·cm^−3^), H is the soil horizon thickness (cm), and δ is the fraction (%) of gravels >2 mm in size in soil. Because the soil gravel size of loess in China is mostly below 2 mm, this fraction was assumed to be 0 [33].

SR values (0–10 cm: 10–40 cm, 0–10 cm: 40–100 cm and 0–10 cm: 100–200 cm) were calculated from the contents of SOC, TN, POC and LOC following the method in Franzluebbers (2002).

CMI values were calculated using following procedures:

Firstly, a C pool index (CPI) was calculated:
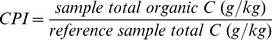
(2)where reference sample is SC soil. Then, a lability index (LI) was calculated:
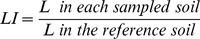
(3)where reference soil is SC soil, and L was calculated from the C lability:
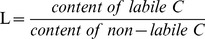
(4)


At last, CMI was calculated:
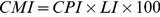
(5)


### Statistical analyses

All statistical analyses were carried out with SPSS 17.0. Analysis of variance (ANOVA) and Duncan's Multiple Range Test (DMRT) at 5% level of significance were used to compare the difference in contents and/or stocks of SOC, TN, POC, LOC, CMI, and SR among different land use types or soil depths. A sample linear-regression analysis was used to estimate the relationships between carbon stocks with CMI or SR values.

## Results

### Changes in contents of SOC, TN, POC and LOC

**Figure 2:**
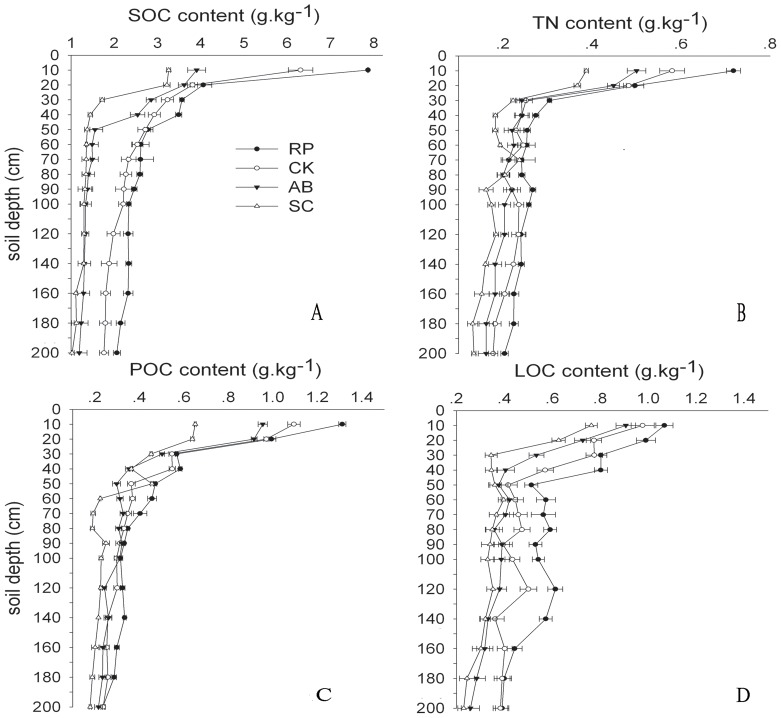
Distribution of soil organic carbon (SOC, A), total nitrogen (TN, B), particulate organic carbon (POC, C), and labile organic carbon (LOC, D) contents of different land used types in soil depth of 0–200 cm. The error bars are the standard errors.

The contents of SOC, TN, POC and LOC responded differently as the change of soil depth (Fig. 2). In all land use types, contents of SOC, TN, POC and LOC in top soil (0–10 cm) were 3.26–7.86 g.kg^−1^, 0.39–0.72 g.kg^−1^, 0.65–1.31 g.kg^−1^ and 0.76–1.07 g.kg^−1^, respectively, which were significantly higher than other soil layers (P<0.05). The contents of SOC, TN, POC and LOC decreased significantly in soil depth of 10–40 cm while the decreases trended to be flatter in subsoil (40–100 cm). Additionally, the differences in contents of SOC, TN, POC and LOC in deep subsoil (100–200 cm) were negligible (P<0.05).

**Figure 3:**
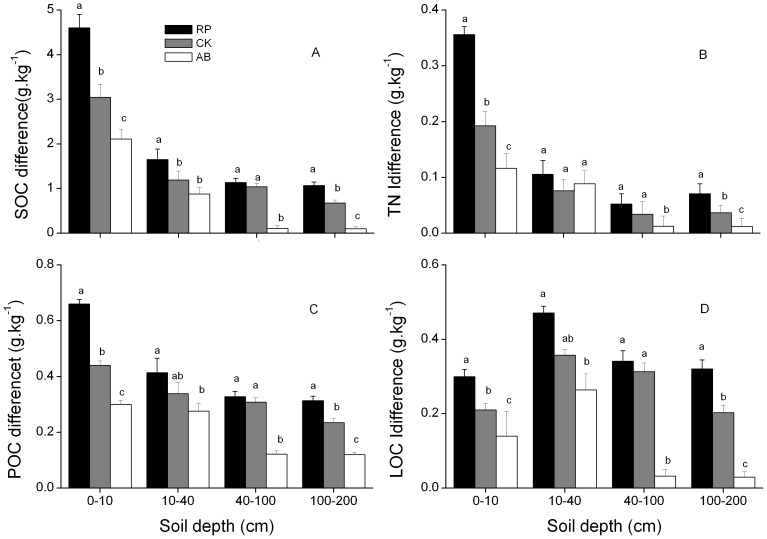
Differences in soil organic carbon (SOC, A), total nitrogen (TN, B), particulate organic carbon (POC, C), labile organic carbon (LOC, D) contents between SC and RP, CK or AB (RP/CK/AB - SC). Error bars are the standard errors. Different lowercase letters indicate significant difference among different land use types within same soil layer (P<0.05). The same for Fig 4

The differences in contents of SOC, TN, POC and LOC between three forest/shrub types (RP, CK and AB) and SC are shown in Fig. 3. The differences in SOC, TN, POC and LOC of RP and SC in soil depths of 0–10 cm and 100–200 cm were significantly higher than that between other land use types and SC (P<0.05). The differences in SOC and TN of RP were 33.78% and 45.97% larger than that of CK, and 54.13% and 67.28% larger than that of AB in soil depth of 0–10 cm (P<0.05), while the differences in POC and LOC were 32.8%, 54.0% higher than that of CK, and 23.3% and 45.0% higher than that of AB (P<0.05). Moreover, the differences in SOC, TN, POC and LOC of RP were 25.05–85.29% higher than that of CK, and 61.78–90.70% higher than that of AB in soil depth of 100–200 cm. Additionally, significant differences in SOC, TN, POC and LOC contents were observed between RP and CK in soil depths of 10–40 cm and 40–100 cm, but there was no difference between CK and AB (P<0.05).

### Changes and distribution of SOC, TN, POC and LOC stocks

**Figure 4:**
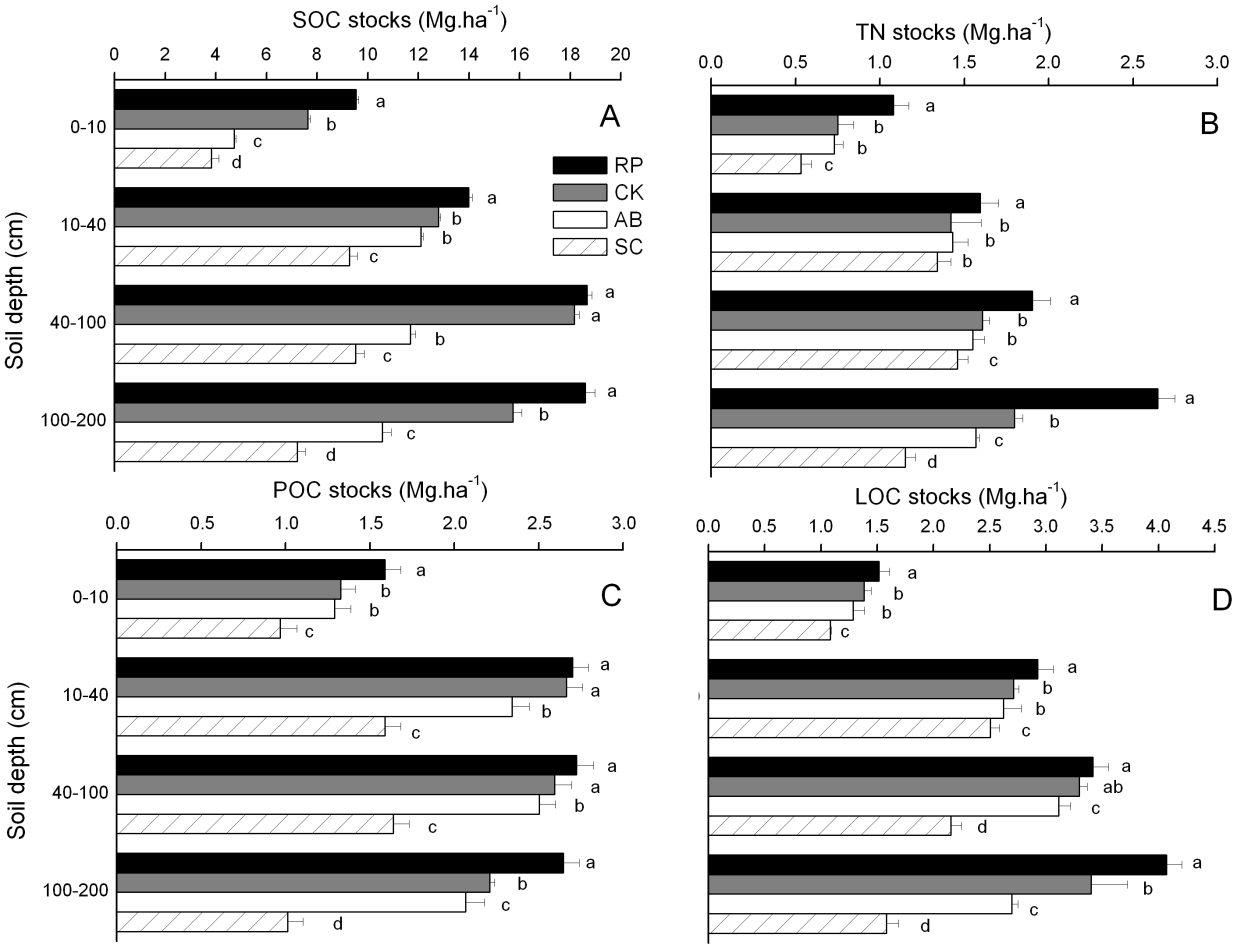
Stocks of soil organic carbon (SOC, A), total nitrogen (TN, B), particulate organic carbon (POC, C), labile organic carbon (LOC, D) of different land use types. The error bars are the standard errors.

SOC, TN, POC and LOC stocks of RP, CK and AB were higher than SC in all soil profiles (Fig 4). The SOC, TN, POC and LOC stocks of RP were significantly increased (P<0.05), which were 0.43–5.8 Mg.ha^−1^, 0.25–4.70 Mg.ha^−1^, 0.44–9.14 Mg.ha^−1^ and 1.49–11.38 Mg.ha^−1^ higher than that of SC in soil layers of 0–10, 10–40, 40–100 and 100–200 cm, respectively. Moreover, the stocks of SOC, TN, POC and LOC in soil layer of 100–200 cm of RP were higher than that of CK and AB by 15.4–32.1% and 21.8–43.1%, respectively (P<0.05).

**Figure 5:**
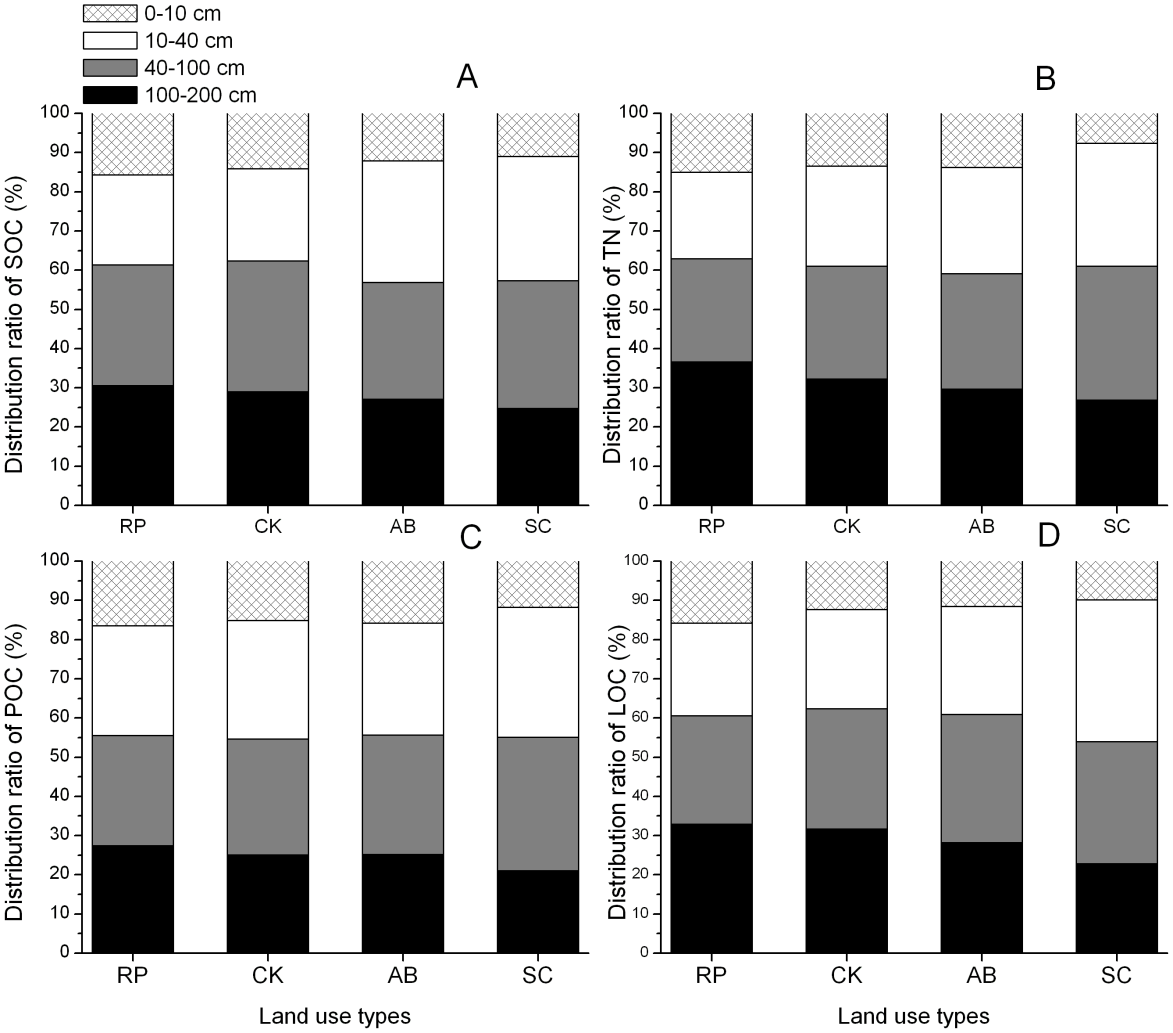
Distribution ratios of soil organic carbon (SOC, A), total nitrogen (TN, B), particulate organic carbon (POC, C), labile organic carbon (LOC, D) in soil depth of 0–200 cm under different land use types.

The SOC, TN, POC and LOC stocks responded differently as the change of soil depth (Fig. 5). Although the distribution of SOC, TN, POC and LOC stocks in soil depths of 0–10 cm and 10–40 cm accounted for the majority, 26.36–34.06% and 21.08–36.62% were **distributed** in soil layers of 40–100 cm and 100–200 cm, respectively. Among four land use types, the highest proportion of SOC, TN, POC and LOC stocks were found in RP, while the lowest were in soil depths of SC in 0–10 cm and 100–200 cm of SC. The proportion of SOC, TN, POC and LOC stocks under RP were higher than SC by 4.68%, 7.32%, 4.65% and 5.96% respectively in soil depth of 0–10 cm soil depth, whereas by 5.90%, 9.78%, 6.30% and 10.06% was higher in soil depth of 100–200 cm soil depth respectively.

Change in SR and CMI values

**Figure 6:**
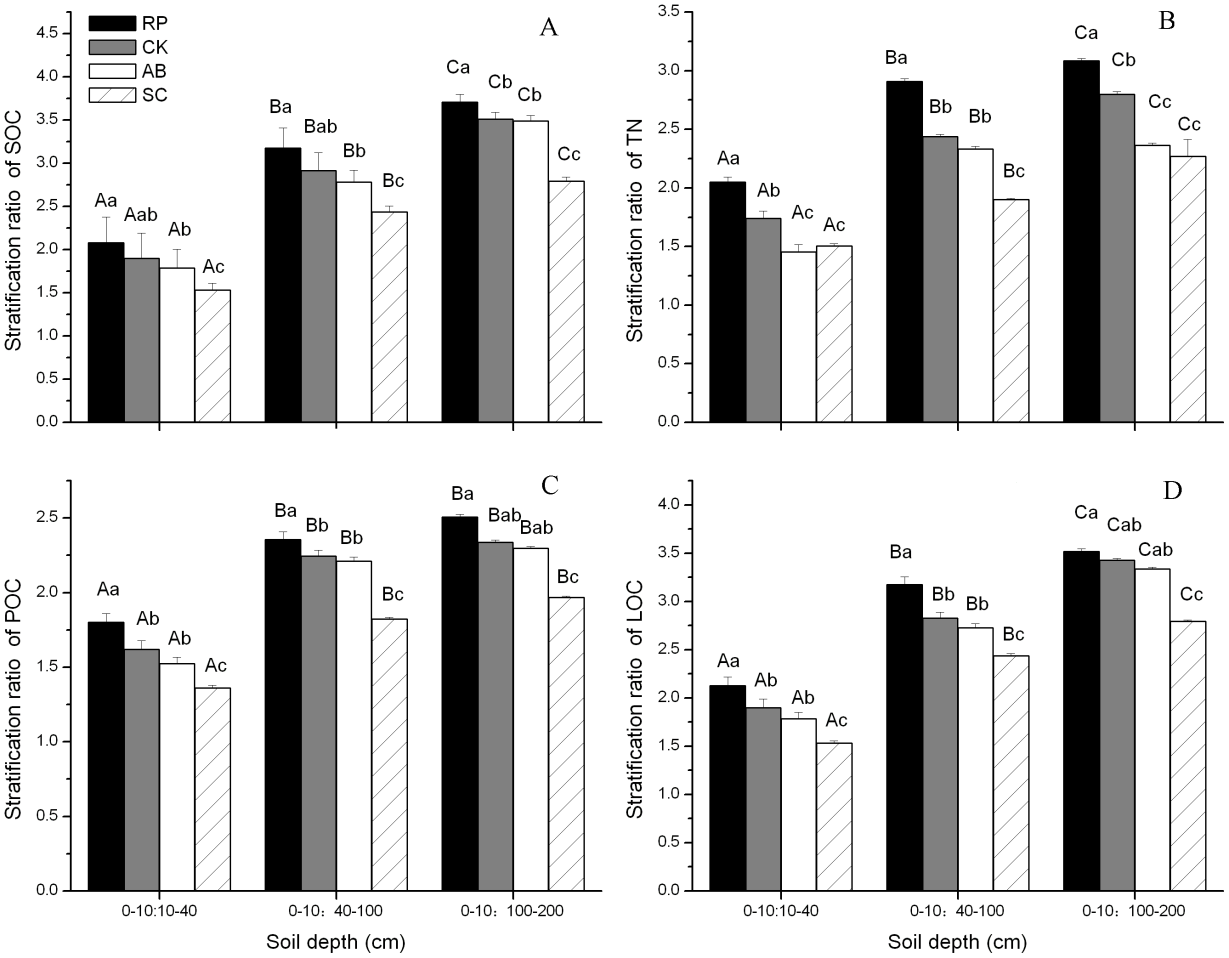
Comparison of stratification ratio of soil organic carbon (SOC, A), total nitrogen (TN, B), particulate organic carbon (POC, C), labile organic carbon (LOC, D) under different land use types. Different uppercase letters indicate significant difference among different soil depths within same land use type while the different lowercase letters indicate significant difference among different land use types within same soil depth. The error bars are the standard errors.

Responses of SR in different land use types to change of soil depth were different (Fig 6). The SR values of SOC, TN and LOC differed significantly among different soil depths (P<0.05), while the SR values of LOC differed only between 0–10:10–40 cm, 0–10:40–100 cm and 0–10:100–200 cm. Among four land use types, the SR values of SOC, TN, POC and LOC of RP were the highest, but that of SC were the lowest in each soil depth(P<0.05). The SR values of SOC, TN, POC and LOC were in a decreasing order of CK>AB>SC. The SR values differed significantly between CK or AB with SC (P<0.05), while there was no significant difference between CK and AB. Additionally, the ratios of SR values of SOC, TN, POC and LOC in the surface layer (0–10 cm) to that in layer of 10–40 cm were >2.0 in most cases.

**Figure 7:**
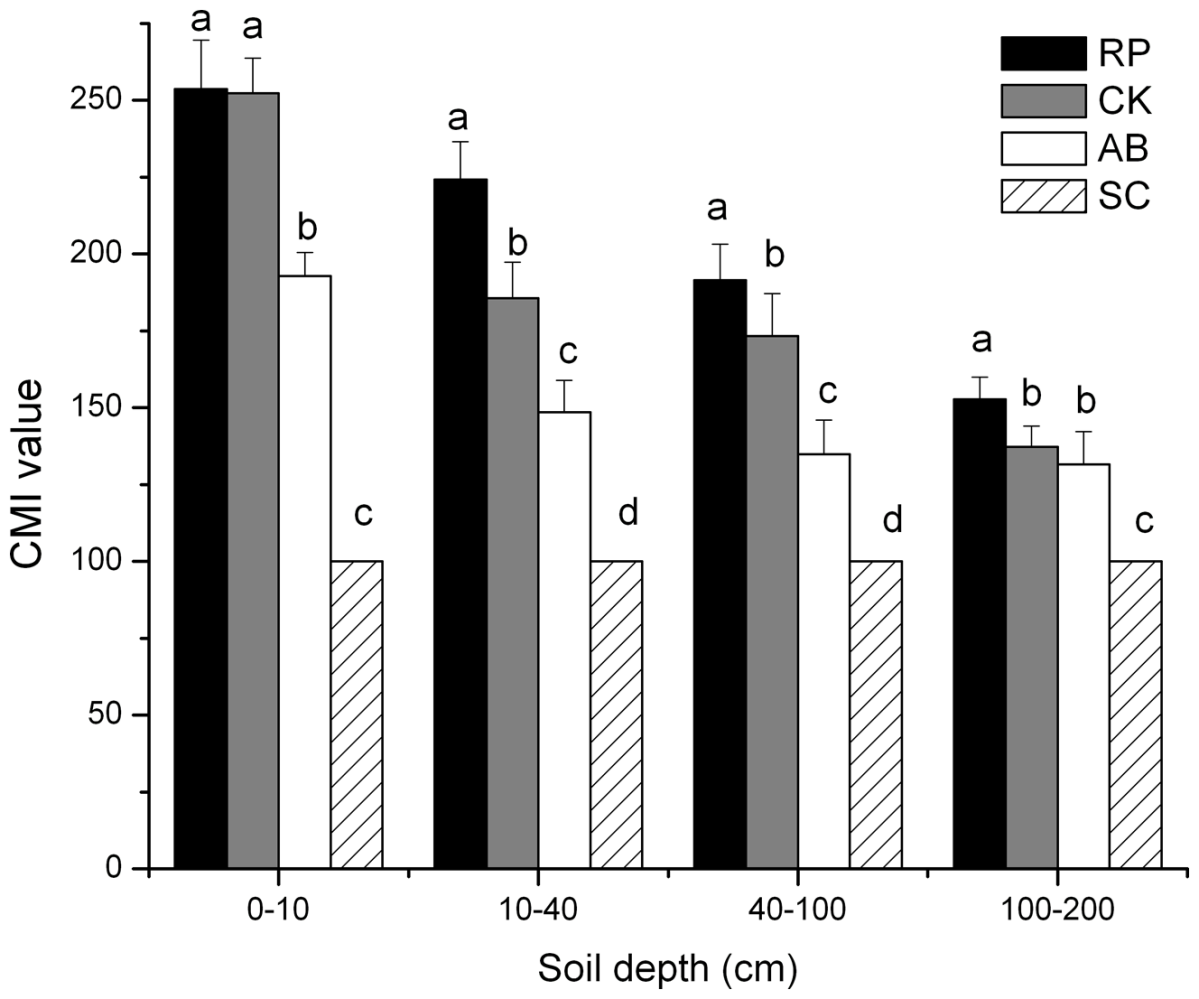
Carbon management index (CMI) values of different land use types at different soil depths. The error bars are the standard errors. Different lowercase letters indicate significant difference among different land use types within same soil depth.

The CMI values were significantly affected by land use types. In our study, the CMI values were in a decreasing order of RP>CK>AB>SC in four soil profiles and CMI values were significantly enhanced by RP compared with SC (Fig 7). Averaged CMI values of RP, CK, and AB were 40.60%, 50.54%, 37.81%, and 14.1% higher than that of SC in soil layers of 0–10 cm, 10–40 cm, 40–100 cm and 100–200 cm.

Regression equations to assess CMI/SR values of TN, POC, and LOC (Y) were showed in Table 2. There was a significant positive correlation between CMI/SR values of TN, POC, and LOC with SOC stocks in surface soil and deep soil.

**Table 2:** Regression equations among SOC stocks and CMI/SR for different soil layers.

Axis		Soil depth (cm)	Equations	R^2^	Significant level
X, CMI/SR^a^	Y, SOC stocks^b^				
CMI	SOC	0–10	Y = 53.5+23.20X	0.91	P = 0.048
		10–40	Y = −146+25.81X	0.97	P = 0.027
		40–100	Y = 24.2+8.60X	0.93	P = 0.023
		100–200	Y = 77.5+4.05X	0.93	P = 0.046
TN	SOC	0–10:10–40	Y = 1.12+0.08X	0.93	P = 0.047
		0–10:40–100	Y = 1.26+0.07X	0.96	P = 0.032
		0–10:100–200	Y = 1.67+0.07X	0.98	P = 0.017
POC	SOC	0–10:10–40	Y = 1.18+0.06X	0.97	P = 0.023
		0–10:40–100	Y = 1.55+0.04X	0.93	P = 0.041
		0–10:100–200	Y = 1.74+0.04X	0.92	P = 0.047
LOC	SOC	0–10:10–40	Y = 1.30+0.08X	0.96	P = 0.031
		0–10:40–100	Y = 1.95+0.05X	0.97	P = 0.025
		0–10:100–200	Y = 2.53+0.05X	0.98	P = 0.013

a CMI =  carbon management index, SR =  stratification ration, TN =  SR of total nitrogen, POC =  SR of particulate organic carbon, LOC =  SR of labile organic carbon.

b For the Y-axis, the SOC stocks (0–10 cm, 40–100 cm, and 100–200 cm) were used to analyze correlations between SOC stocks and SR values of TN, POC, and LOC (0–10:10–40, 0–10:40–100, 0–10:100–200).

## Discussion

### SOC, TN, POC and LOC contents and SOC and TN stocks

Vegetation can greatly influence soil quality, C and N cycling, and regional socioeconomic development [34]–[35]. It is also reported that converting cropland into land with perennial vegetation would increase the SOC content [36]. Our results showed that land use type and soil depth significantly affected the contents of SOC and TN (Fig. 2). The conclusion that both land use type and soil depth are important factors influencing the soil carbon and nitrogen distribution was consistent with previous studies [35], [37]. We also observed that the lowest SOC, TN, POC and LOC contents were found in slope cropland (Fig 4), which essentially agree with a previous study [38], indicating that the conversion of slope cropland to vegetation improves the C and N contents. A possible reason is that the lower residue input into the soil in slope cropland leads to lower SOC and TN contents. Additionally, our results showed that SOC, TN, POC and LOC contents of RP were greater than that of CK and AB (Fig 4). It infers that the effects of RP on soil C and N play a significant role in land use and ecosystem management. The conclusion was consistent with Qiu et al [39], who reported that RP has potential to improve SOC content in the loessial gully region of the Loess Plateau and the improvements are greater in long-term than middle-term.

Recently it was reported that the depth of sampling is an important factor for the measurement of change in SOC stocks [40], and land use could influence subsoil C pools [41]. We found that SOC, TN, POC and LOC stocks of RP, CK, and AB were higher than SC for different soil profiles, especially in depths of 40–100 cm and 100–200 cm (Fig. 4 and 5). It is demonstrated that converting slope cropland into woodland and shrubland not only affects SOC and TN stocks in surface soil, but also largely influences that in deep soil. The result was consistent with Wang et al [42], who reported that deep layer (50–200 cm) SOC stocks were equivalent to approximately 25% of that in the shallow layer (0–50) in Hilly Loess Plateau. This is mainly due to the fact that SOC input into subsoil is largely affected by plant roots and root exudates, dissolved organic matter and bioturbation. In addition, most important factors leading to protection of SOC in subsoil include the spatial separation of SOM, microorganisms and extracellular enzyme activity related to the heterogeneity of C input [43]. As a result, stabilized SOC in subsoil is horizontally stratified.

### Stratification ratios of SOC, TN, POC, and LOC

According to Franzluebbers [7], SOC SR values >2 in degraded conditions is uncommon, and the SR values of SOC are generally low and seldom reach 2.0. And SR values of soil organic C and N pools with value of >2 would be an indicator that soil quality might be improved [7]. In our study, the most of SR in SOC, TN, POC and LOC was more than 2 after convert slope cropland to forest or shrub land (Fig. 6). This means soil quality was improved in these afforested soils without disturbance. Greater C stratification ratios could be related to the fact that, during soil recovery by re-vegetation or land abandonment, soil was undisturbed thus reducing oxidation and favoring soil C [44]. The result was consistent with Sá et al [45]. Similar results were also reported by Moreno et al [46] and Franzluebbers [7], who reported that stratification of SOC occurs over time when soil tillage and disturbance is stopped and it is usually greater in undisturbed soils than in disturbed soils. In addition, the stratification may increase with time, and SOC, TN, POC and LOC contents are still aggrading but have not reached soil C saturation yet. That is the reason why SR values of SOC, TN, POC and LOC under CK and AB were higher but no significant differences were observed compared with SC (Fig. 6). Sá et al [45] concluded that the SOC pool stabilization may be attained in about 40 years after long-term no-tillage adoption.

### Carbon management index

CMI value was calculated to obtain indications of the C dynamics of the system and provide an integrated measure for quantity and quality of SOC [15]. Soils with higher CMI values are considered as better managed [47]. We found that CMI values were significantly enhanced by RP forest compared with CK, AB and SC in both surface soil and subsoil and deep soil (Fig. 7). Soil management under RP plot was more appropriate to improve the SOC status than other land use types. Similar result was reported by Qiu et al [39], who illustrated that RP forest has significantly increased SOC, total nitrogen, ratio of carbon to nitrogen and ratio of carbon to phosphorus compared to other vegetation types. Our result showed that there were significant positive correlations between SOC stocks and CMI/SR in both surface soil and deep soil (Table 2). These findings showed that SR values of SOC, TN, POM, and LOC, and CMI are suitable indicators for evaluating soil quality and C changes induced by GTGP in surface soil and deep soil.

## Conclusion

In this study, the SOC, TN, POC and LOC contents of RP, CK and AB in soil layer of 100–200 cm were higher than SC, especially for RP plot. Although the SOC, TN, POC and LOC stocks in soil layer of 100–200 cm were lower, there was more than 27.38–36.62%, 25.10–32.91%, 21.59–31.69% and 21.08–26.83% of SOC, TN, POC and LOC stocks were distributed in 100–200 cm soil depth under RP, CK and AB. Meanwhile, the SR of SOC, TN, POC and LOC in the surface to lower depth ratio (i.e., 0–10:10–40 cm) was >2.0 in most of case. And SR and as well CMI values were significantly enhanced by RP compared with SC in deep soil (100–200 cm) (P<0.05). Indicating that soil quality was improved after converting slope land into perennial vegetation, especially under RP plot from surface soil to deep soil. Moreover, there were significant and positive correlations between SOC stocks and CMI or SR of TN, POC, LOC both surface soil and deep soil indicated that the SR and CMI value are suitable indicators for evaluating soil quality and C changes in surface soil as well as in deep soil. We, therefore, propose deep soil organic carbon should be considered in C cycle induced by Grain-to-Green Program (GTGP) and under RP forest is more appropriate strategy to improve the SOC status than other land use types in surface soils and deep soil.
